# Low accuracy of endoscopic ultrasonography for detailed T staging in gastric cancer

**DOI:** 10.1186/1477-7819-10-190

**Published:** 2012-09-15

**Authors:** Han Hong Lee, Chul Hyun Lim, Jae Myung Park, Yu Kyung Cho, Kyo Young Song, Hae Myung Jeon, Cho Hyun Park

**Affiliations:** 1 Department of Surgery, Division of Gastrointestinal Surgery, College of Medicine, The Catholic University of Korea, Seoul, South Korea; 2 Department of Internal Medicine, College of Medicine, The Catholic University of Korea, Seoul, South Korea

**Keywords:** Cancer staging, Endoscopic ultrasonography, Stomach neoplasm

## Abstract

**Background:**

The accuracy of endoscopic ultrasonography (EUS) for preoperative staging of gastric cancer varies. The aim of this study was to investigate the accuracy of EUS tumor (T) and node (N) staging, and to identify the histopathological factors influencing accuracy based on the detailed tumor depth of gastric cancer.

**Methods:**

In total, 309 patients with gastric cancer with confirmed pathological staging underwent EUS examination for preoperative staging at Seoul St. Mary’s Hospital, Korea, between January and December 2009. The T and N staging of EUS and the pathologic report were compared.

**Results:**

The overall accuracies of EUS for T stage and the detailed T stages were 70.2% and 43.0%, respectively. In detailed stage, tumors greater than 50 mm in diameter were significantly associated with T overstaging (odds ratio (OR) = 2.094). The overall accuracy of EUS for N staging was 71.2%. Tumor size (20 mm ≤ size < 50 mm, OR = 4.389; and 50 mm ≤ size, OR = 8.170), cross-sectional tumor location (circumferential, OR = 4.381) and tumor depth (submucosa, OR = 3.324; muscular propria, OR = 6.923; sub-serosa, OR = 4.517; and serosa-exposed, OR = 6.495) were significant factors affecting incorrect nodal detection.

**Conclusions:**

Careful attention is required during EUS examination of large-sized gastric cancers to increase accuracy, especially for T staging.

## Background

Gastric cancer is the second leading cause of cancer deaths worldwide [1]. Removal of a primary tumor by perigastric lymphadenectomy is accepted as the only way to increase long-term survival in patients with gastric cancer [2]; however, the operative method and access route can vary, based on preoperative tumor stage and tumor characteristics. Compared with serosal-exposed lesions of with a tumor (T) stage of greater than T3, which can be distinguished somewhat easily, distinguishing preoperative tumor depths for gastric cancers less than T2 is crucial because it determines the operative method, including choice of endoscopic submucosal dissection (ESD), laparoscopic gastrectomy, or open gastrectomy. In addition, preoperative prediction regarding the presence of lymph-node metastasis is a decisive factor in selecting endoscopic or surgical resection.

The development of endoscopic ultrasonography (EUS) has increased the accuracy of preoperative staging and diagnosis of upper gastrointestinal malignancies, including gastric cancer, and has had a major effect in determining the therapeutic options for gastric cancer. At present, EUS is the most valuable method for T staging of gastric cancer and is also used for detecting regional lymph-node involvement [3-6]; however, the accuracy of EUS for T and perigastric N staging varies [6,7]. In addition, there have been few studies assessing the accuracy rate of T staging with regard tothe gastric wall layers (mucosa, submucosa, muscular propria, sub-serosa, and serosa), which can be identified by EUS. Consequently, there are no guidelines regarding the clinicopathological factors requiring attention during a EUS check-up, and the accuracy rates are still undetermined.

The aim of this study was to determine the accuracy of EUS with regard to tumor depth and nodal metastasis, and to analyze the histopathological factors affecting the accuracy of EUS in gastric cancer, with a particular focus on the detailed gastric wall layers.

## Methods

### Ethics approval

The study protocol was approved by the institutional review board (KC10RISE0441).

### Patients

From January to December 2009, 491 patients underwent gastric cancer surgery at Seoul St. Mary’s Hospital, Seoul, Korea.We prospectively collected the clinicopathological date of 436 patients who were diagnosed with pathological cancer staging (the remaining patients comprised thirty-seven patients who could not undergo gastric resection, sixteen patients for whom preoperative cancer staging was difficult (including incomplete ESD, neoadjuvant chemotherapy, and remnant gastric cancer) and two patients with pathological non-measurable lesions). Of these 436 patients, 309 (184 men (59.5%), 125 women (40.5%); mean ± SD age 57.5 ± 12.2 years, range 26–86 years),for whom EUS was used as a preoperative diagnostic tool, were enrolled in this retrospective study.

### Endoscopic ultrasonography equipment and technique

**Figure 1:**
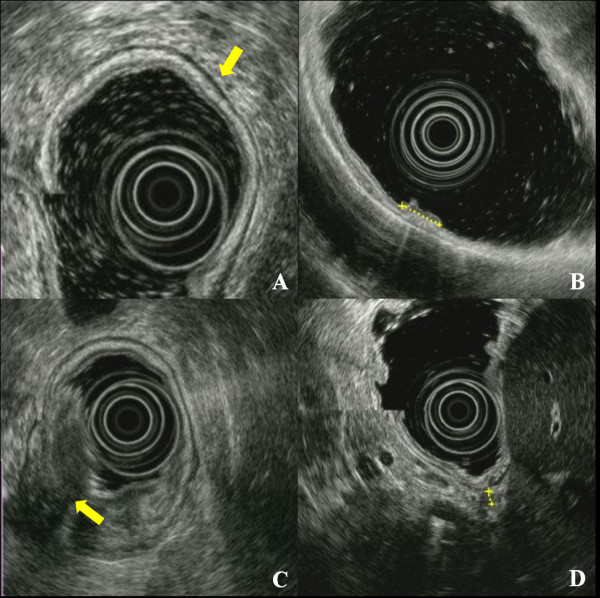
**Endoscopic ultrasonography (EUS) (A) EUS showing normal layers of gastric wall, with the five-layered echo pattern being clearly visible (arrow).** (**B**) EUS of early gastric cancer (T1a), showing a focal thickening area confined to layers 1 and 2(dotted line). (**C**) EUS image of advanced gastric cancer (T3); the tumor involves all layers and extends beyond the the outermost layer of the gastric wall (arrow). (**D**) EUS showing a metastatic lymph node (dotted line).

EUS was performed with a radial transducer (12 to 20 MHz; GF-UM2000; Olympus, Tokyo, Japan), and in some cases, a 20 MHz miniprobe (Olympus) was also used. After the stomach was filled with 300 to 600 mL of water, the tumor infiltration depth was evaluated based on the five-layered gastric wall structure. Assessment of tumor depth by EUS was made in accordance with the International Union Against Cancer TNM Classification (6th edition): T1a, cancer invading the mucosal layer; T1b, cancer invading the submucosal layer; T2a, cancer invading the muscularis propria; T2b, cancer invading the subserosal layer; T3, cancer penetrating the serosal layer; and T4, cancer invading adjacent structures (Figure 1).Lymph nodes were defined by the size and shape of identified hypoechoic structures in perigastric tissues. Hypoechoic nodes larger than 5 mm with round and well-demarcated margins were considered positive for metastasis. A diagnosis of N2 stage was made when the lymph-node metastasis was > 30 mm from the primary lesion. Fine-needle aspiration (FNA) was not performed for lymph nodes that appeared to be metastatic.

### Assessment of endoscopic ultrasonography staging

The preoperative EUS T and N staging results were compared with each pathological stage. The accuracy, overstaging, and understaging rates for EUS T staging were calculated using two methods: all stages (T1, T2, T3, and T4) and all detailed stages (T1a, T1b, T2a, T2b, T3, and T4).The correlations between EUS T overstaging and understaging and histopathological factors (including tumor size, tumor location, histological type, ulceration, and Lauren’s classification,) were analyzed for the 309 cases. Tumor size was classified as less than 20 mm, 2 to 50 mm, or greater than 50 mm. Tumor locations were subclassified using two criteria. One group was divided into upper, middle, and lower thirds, based on the longitudinal portions of the stomach, and the other group was divided into wall, curvature, and circumferential, sorted according to the cross-sectional portions. The histological types of gastric cancer, in accordance with the World Health Organization classification, were categorized into differentiated and undifferentiated types. Poorly differentiated tubular adenocarcinoma, signet ring cell adenocarcinoma, and mucinous adenocarcinoma belonged to the undifferentiated group. The accuracy rate for EUS N staging And the correlations between EUS N accuracy and histopathological factors that added to tumor depth were analyzed for the 309 cases.

### Statistical analysis

Tumor characteristics and their EUS stages were analyzed using the χ^2^ test or Fisher’s exact test for proportions. Factors with an effect on EUS TN staging were analyzed by binary multiple regression analysis. Statistical analyses were performed with SPSS software (version 13.0; SPSS, Chicago, IL, USA), and *P <* 0.05 was considered significant.

## Results

### Study groups

Of the 309 patients, the pathological T staging showed that there were 107 cases of T1a (40.8%), 85 T1b (32.4%), 36 T2a (13.7%), 34 T2b (13.0%), 45 T3 (14.6%), and 2 of T4 (0.6%). For pathological N stage, there were 213 N0 cases, which comprised the largest proportion (68.9%). The clinical data and pathological stage of each group are shown in Table 1.

**Table 1:** Patient demographics and pathological stages

**Variable**	**Result (*n* = 309)**
Age, years^1^	57.5 ± 12.2 (26–86)
Gender	
Male	184 (59.5)
Female	125 (40.5)
pT	
T1a	107 (34.6)
T1b	85 (27.5)
T2a	36 (11.7)
T2b	34 (11.0)
T3	45 (14.6)
T4	2 (0.6)
pN	
N0	213 (68.9)
N1	72 (23.3)
N2	11 (3.6)
N3	13 (4.2)
pM	
M0	301 (97.4)
M1	8 (2.6)
Sixth AJCC stage	
Ia	167 (54.0)
Ib	60 (19.4)
II	38 (12.3)
IIIa	22 (7.1)
IIIb	6 (1.9)
IV	16 (5.2)

AJCC, American Joint Committee on Cancer.

^1^Values are means ± standard deviation (range). All other values are n (%).

### T staging by endoscopic ultrasonography

In all 309 cases examined by EUS, the overall accuracy of EUS for T staging was 70.2% (217/309). The rates of overstaging and understaging were 23.6% (73/309) and 6.1% (19/309), respectively. In the detailed T staging of 309 cases, the overall accuracy rate decreased to 43.0% (133/309), and the overstaging and understaging rates increased to 44.3% (137/309) and 12.6% (39/309), respectively. Two versions of the comparison of T stage by EUS and pathology are shown in Table 2.

**Table 2:** Accuracy of endoscopic ultrasonography (EUS) for T staging

**EUS stage**	**Pathological stage**					**EUS stage**	**Pathological stage**						
	**T1**	**T2**	**T3**	**T4**	**Total**		**T1a**	**T1b**	**T2a**	**T2b**	**T3**	**T4**	**Total**
T1	155	9	3	0	174	T1a	34	11	0	0	0	0	46
T2	36	37	5	0	87	T1b	60	50	6	3	3	0	128
T3	1	22	25	2	45	T2a	11	21	16	9	2	0	64
T4	0	2	12	0	3	T2b	2	2	4	8	3	0	23
						T3	0	1	9	13	25	2	45
						T4	0	0	1	1	12	0	3
Total	192	70	45	2	309	Total	107	85	36	34	45	2	309
Overall accuracy, %			70.2				43.0						
Overall accuracy, % ^1^			–				41.2						

^1^pT1a–pT2b; *n =* 262.

T overstaging was correlated with tumor size (*P =* 0.03 however, T understaging was not related to any clinicopathological factor. In multivariate analysis, a tumor size greater than 50 mm was a significant factor correlated with overstaging by EUS (odds ratio (OR) = 2.09; 95% confidence interval (CI) 1.13 to 3.88, *P <* 0.02) (Table 3; Table 4). Subgroup analysis was performed, and the cases were divided into two groups based on tumor depth for overstaging and understaging: early gastric cancers (EGCs; T1a, T1b) and advanced gastric cancers (AGCs; T2a, T2b, T3, T4). Tumor size was correlated with EUS overstaging in both subgroups (*P =* 0.03, *P <* 0.001, respectively), and cross-sectional tumor location (*P =* 0.04) was associated with overstaging in the AGC group. In multivariate analysis for subgroup, a tumor size greater than 50 mm was also a factor affecting EUS overstaging in the EGC (OR = 3.00, 95% CI, 1.19 to 7.56; *P =* 0.02) and AGC (OR = 5.40; 95% CI, 2.26 to 12.91; *P <*0.01) subgroups (Table 4; Table 5). No factors were significantly associated with understaging in subgroup analysis (data not shown).

**Table 3:** Histopathological factors affecting endoscopic ultrasonography (EUS) T staging

**Variables**	***n***	**Overstaging, %**	***P***	**Understaging, %**	***P***
Total	309	137 (44.3)		39 (12.6)	
Tumor size, mm					
<20	80	30 (37.5)	0.03	7 (8.8)	0.42
2–50	141	58 (41.1)		21 (14.9)	
≥50	88	49 (55.7)		11 (12.5)	
Tumor locationI					
Upper	18	6 (33.3)	0.62	3 (16.7)	0.28
Middle	137	61 (44.5)		21 (15.3)	
Lower	154	70 (45.5)		15 (9.7)	
Tumor locationII					
Wall	162	70 (43.2)	0.32	20 (12.3)	0.65
Curvature	131	57 (43.5)		16 (12.2)	
Circumferential	16	10 (62.5)		3 (18.8)	
Histological type					
Differentiated	147	66 (44.9)	0.85	21 (14.3)	0.40
Undifferentiated	162	71 (43.8)		18 (11.1)	
Ulceration					
Yes	227	94 (41.4)	0.08	31 (13.7)	0.36
No	82	43 (52.4)		8 (9.8)	
Lauren					
Intestinal type	142	63 (44.4)	0.88	16 (11.3)	0.15
Mixed type	60	25 (41.7)		12 (20.0)	
Diffuse type	107	49 (45.8)		11 (10.3)	

**Table 4:** Multivariate analysis of histopathological factors affecting EUS T staging

**Variable**	***P***	**Odds Ratio**	**95% CI**
Overstaging			
Tumor size ≥ 50 mm	0.020	2.09	1.13 to 3.88
EGC overstaging			
Tumor size ≥ 50 mm	0.02	3.00	1.19 to 7.56
AGC overstaging			
Tumor size ≥ 50 mm	< 0.001	5.40	2.26 to 12.91

AGC, advanced gastric cancer; CI, confidence interval; EGC, early gastric cancer; EUS, endoscopic ultrasonography.

**Table 5:** Histopathological factors affecting T overstaging in the subgroups

**Variables**	**EGCs**			**AGCs**		
	***n***	**Overstaging, n (%)**	***P***	***n***	**Overstaging, n (%)**	***P***
Total	192	97 (50.5)		117	40 (34.2)	
Tumor size, mm						
< 20	75	30 (40.0)	0.03			
2to50	90	49 (54.4)		56	9 (16.1)^1^	< 0.001
≥ 50	27	18 (66.7)		61	31 (50.8)	
Tumor locationI						
Upper	3	1 (33.3)	0.87	15	5 (33.3)	
Middle	81	42 (51.9)		56	19 (33.9)	0.99
Lower	108	54 (50.0)		46	16 (34.8)	
Tumor locationII						
Wall	115	57 (49.6)	0.88	47	13 (27.7)	
Curvature	75	39 (52.0)		56	18 (32.1)	
Circumferential	2	1 (50.0)		14	9 (64.3)	0.04
Histological type						
Differentiated	102	47 (46.1)	0.19	45	19 (42.2)	0.15
Undifferentiated	90	50 (55.6)		72	21 (29.2)	
Ulceration						
Yes	139	68 (48.9)	0.47	88	26 (29.5)	0.07
No	53	29 (54.7)		29	14 (48.3)	
Lauren						
Intestinal type	101	46 (45.5)	0.33	41	17 (41.5)	0.32
Mixed type	31	18 (58.1)		29	7 (24.1)	
Diffuse type	60	33 (55.0)		47	16 (34.0)	

EGCs*,* early gastric cancers; AGCs, advanced gastric cancers.

^1^0to50 mm.

### N staging by endoscopic ultrasonography

The comparative results of N staging by EUS and the pathological reports are shown in Table 6. The N staging accuracy in the 309 patients was 71.2%. Incorrect nodal staging by EUS was correlated with tumor size (*P <* 0.001), cross-sectional tumor location (*P <* 0.001), and tumor depth (*P <* 0.001), and these three factors were significantly associated with inaccurate nodal staging on multivariate analysis. Tumor size greater than 50 mm was the strongest factor for inaccurate EUS nodal staging (OR = 8.170, 95% CI 2.47 to 27.05, *P =* 0.001), and a tumor size greater than 20 mm was also a significant factor (OR = 4.389, 95% CI 1.45 to 13.31, *P <* 0.010). Circumferential location was identified as a significant factor (OR = 4.38, 95% CI 1.02 to 18.83, *P <* 0.05), and all tumor depths over the mucosal layer were significant factors for inaccurate nodal staging (submucosa, OR = 3.324, 95% CI 1.35 to 8.20, *P <* 0.01, muscular propria, OR = 6.92, 95% CI 2.52 to 19.03, *P <* 0.001, sub-serosa, OR = 4.52, 95% CI 1.57to12.10, *P <* 0.01, serosa-exposed, OR = 6.50, 95% CI 2.28to 18.51, *P <*0.001) (Table 7).

**Table 6:** Accuracy of endoscopic ultrasonography (EUS) for N staging

**EUS stage**	**Pathological stage**				
	**N0**	**N1**	**N2**	**N3**	**Total**
N0	190	44	1	1	236
N1	22	27	7	10	66
N2	1	1	3	2	7
Total	213	72	11	13	309
Overall accuracy, %					

**Table 7:** Histopathological factors affecting endoscopic ultrasonography (EUS) N staging

**Variables**	***n***	**Incorrect staging, n (%)**	***P***	**Odds ratio (95% CI)**	***P***
Total	309	90 (29.1)			
Tumor size, mm					
< 20 mm	80	4 (5.0)	< 0.001	4.389 (1.447to13.307)	< 0.01
2 to 50 mm	141	38 (27.0)		8.170 (2.468to27.050)	0.001
≥ 50 mm	88	48 (54.5)			
Tumor location I					
Upper	18	9 (50.0)	0.13		
Middle	137	38 (27.7)			
Lower	154	43 (27.9)			
Tumor location II					
Wall	162	38 (23.5)	< 0.001	1.00 (0.55to1.82)	0.10
Curvature	131	39 (29.8)		4.38 (1.02to18.83)	0.05
Circumferential	16	13 (81.3)			
Histological type					
Differentiated	147	41 (27.9)	0.65		
Undifferentiated	162	49 (30.2)			
Ulceration					
Yes	227	63 (27.8)	0.38		
No	82	27 (32.9)			
Lauren					
Intestinal type	142	38 (26.8)	0.23		
Mixed type	60	23 (38.3)			
Diffuse type	107	29 (27.1)			
Tumor depth					
Mucosa	107	8 (7.5)		3.32 (1.35to8.20)	< 0.01
Sub-mucosa	85	22 (25.9)		6.92 (2.52to19.03)	< 0.001
Muscular propria	36	16 (44.4)		4.52 (1.57to12.10)	< 0.01
Sub-serosa	34	15 (44.1)		6.49 (2.28to18.51)	< 0.001
Serosa-exposed	47	29 (61.7)	< 0.001		

CI, confidence interval.

## Discussion

The accuracy of EUS for gastric cancer varies. In previous studies, EUS has had an accuracy of 65% to 92% for T staging, and 50% to 90% for N staging [6-13]. In most studies, T stage has been classified as T1, T2, T3, or T4, instead of T1a, T1b, T2a, T2b, T3, and T4 [3-5,9-27]. A few studies have estimated the accuracy of detailed T staging by EUS, but they were limited to distinguishing the mucosa from the submucosal layer [8,28-30]. In the present study, we found the overall accuracy of EUS for T staging was 70.2%, which was similar to previous studies. However, when T stage was subdivided into the detailed stages, the overall accuracy rate for T staging was only 43.0%, and the overstaging rate increased sharply to 44.3%. These unexpected results were mainly the result of overstaging of mucosal lesions into submucosal lesions, and occurred in 60 cases.

**Figure 2:**
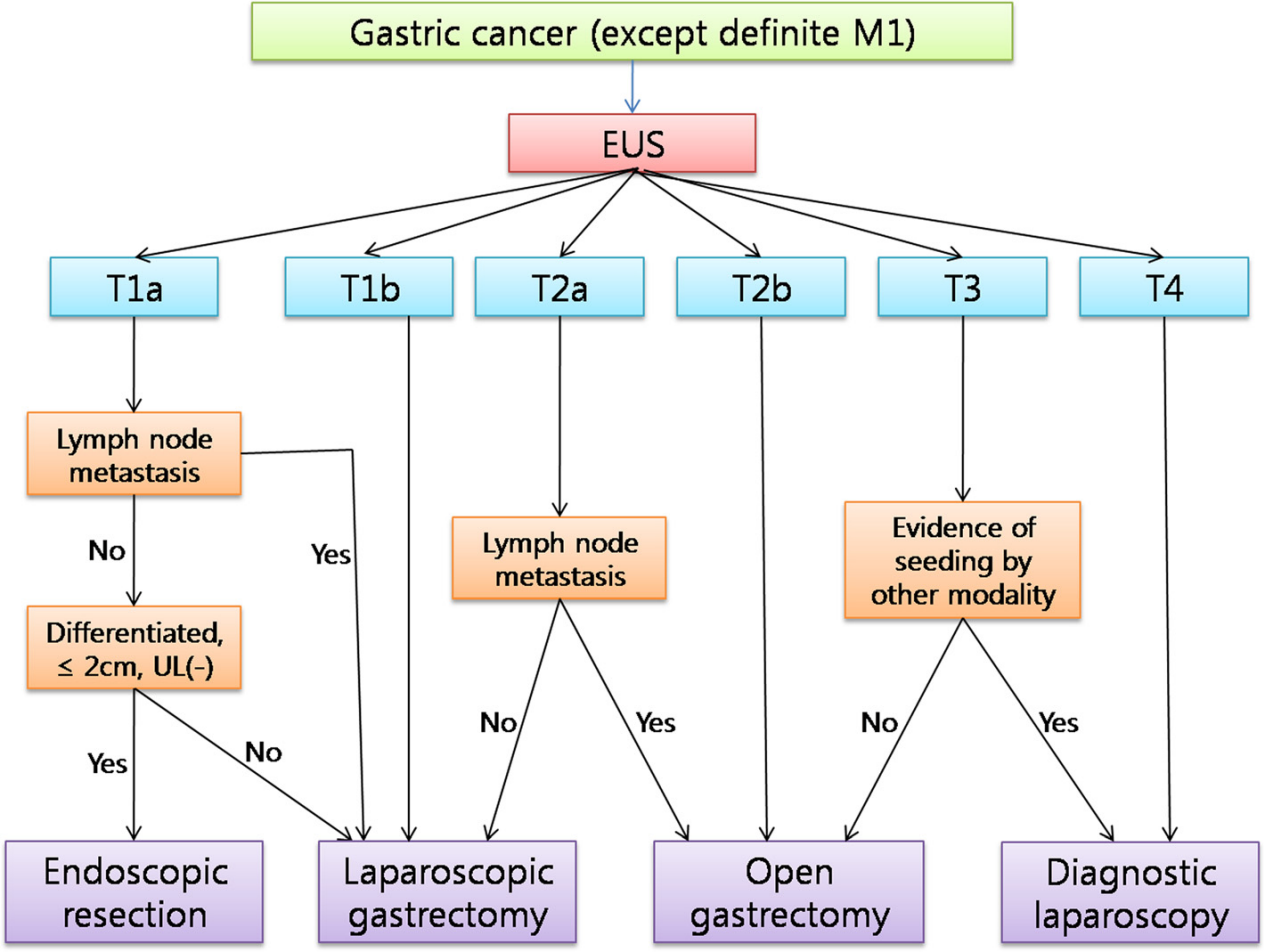
**Treatment algorithm for gastric cancer based on the detailed tumor (T) stages.** EUS, endoscopic ultrasonography; UL, ulceration.

EUS images of the normal gastric wall show a five-layered structure. Gastric cancer invading the serosa can be adequately diagnosed by traditional endoscopy and computed tomography (CT). EUS tends to overestimate the T stage because of the difficulty in distinguishing invasion through the subserosal fat (T2b) and the serosa (T3), which is very thin in some areas [22]. EUS findings for gastric cancer at less than stage T2 can profoundly affect selection of operative method, unlike stages more severe than T3, for which the treatment plan involves open gastrectomy. The treatment algorithm based on the detailed T stage by EUS is described in Figure 2. In our population of 262 cases limited to T2b, the overall accuracy rate decreased to 41.2%, and the overstaging rate increased to 47.7%. Therefore, we attempted to identify the factor(s) affecting the overstaging of depth, and subsequently evaluated the factors in subgroups as EGCs and AGCs.

Our results showed that the rate of overstaging tended to increase with increasing tumor size, and a tumor size greater than 50 mm was a significant factor affecting EUS overstaging. However, the proportion of EGCs (97/137; 70.8%) was greater than that of AGCs (40/137; 29.2%) in cases with overstaging. We performed subgroup analysis to determine whether tumor size, identified as an influencing factor for T overstaging, would work in the same way in both groups with very different proportions. Large tumor size was associated with T overstaging in univariate analysis of both groups, and a tumor size greater than 50 mm was significantly associated. Kim *et al*. [31] reported that tumors larger than 30 mm could cause EUS overstaging. In their study, in which the T1 stage was subdivided into T1a and T1b, but T2 stage was not subdivided, the overall T staging accuracy rate was 71.8%. Of our 309 cases, 171 (55.3%) had tumors larger than 30 mm, with 39% (75/192) were larger than 30 mm in the EGC group. As our results showed a correlation between tumor size and T overstaging, we considered that many tumors greater than 30 mm in size resulted in the low accuracy rate.

Compared with the overall accuracy rate for N staging which was 71.2%, the accuracy rate of the 262 cases with tumor location below the sub-serosa was 76.7%. In contrast to T staging accuracy, the accuracy of N staging increased when we excluded AGCs more severe than T3. This result suggested that tumor depth might influence perigastric nodal detection by EUS, and tumor depth was added as a histopathological factor possibly associated with N staging accuracy. Our results showed that as tumor invasion became deeper, incorrect nodal detection increased as expected, and large-sized tumors were also associated with incorrect EUS nodal staging. The proportion of incorrect nodal staging increased equally to 44% in both the muscular propria and subserosal layers, and these layers (AGC) had higher ORs than the submucosal layer (EGC). T overstaging was correlated with a tumor size greater than 50 mm, whereas incorrect N staging was significantly associated with tumors greater than 20 mm. ESD is currently performed for differentiated, intramucosal lesions of less than 20 mm in diameter without ulcerations, because a tumor size greater than 30 mm, ulcer formation, and lymphatic/vascular involvement are regarded as independent risk factors for lymph-node metastasis in intramucosal cancer [32,33]. However, the expanded ESD criteria include lesions greater than 20 mm in diameter, ulcerative lesions, and minute submucosal cancer. Judging from our results, careful examination of nodal metastases in preoperative EUS staging and cooperative staging with another method, such as CT, is essential for determining the treatment plan for EGC lesions greater than 20 mm in diameter.

Although there was no statistical significance in multivariate analysis, circumferential tumor location was associated with T overstaging of AGCs in subgroup analysis (*P =* 0.04). In addition, this location was a significant factor determining incorrect nodal staging of EUS (*P =* 0.05). A tumor must have a broad surface to occupy a circumferential location within the stomach; therefore, the borderline significance of this particular location was considered as a secondary effect caused by the large size of the tumor.

There are some limitations to our study. First, the examiners who performed the EUS all belong to a single institution. Although many cases were enrolled in our study, the results may be just a reflection of the preference of a single institution. However, all examiners are endoscopic specialists of the upper gastrointestinal tract, and perform more than 100 cases of EUS every year. The second limitation is the definition of EUS N staging. EUS N staging of this study includes the concept of distance and is similar to the nodal staging of Japanese Gastric Cancer Association which is based on the location of metastatic lymph nodes. Because the N staging International Union Against Cancer TNM Classification (6th edition), which was used for pathologic nodal staging in this study, is based on the number of metastatic lymph nodes, the comparison of EUS N staging with pathologic N staging might be inappropriate. Additionally, there is no criterion for N3 staging using EUS.

However, based on our results, we suggest that extra attention should be paid not only to preoperative EUS T staging but to N staging for large-sized EGCs. In addition, because tumors larger than 50 mm, in which the possibility of T overstaging is high, are mostly affiliated with AGCs, and the accuracy of nodal staging tends to decrease with increasing tumor depth, the efficacy of routine preoperative EUS for AGCs should be evaluated. Reddy *et al*. [34] reported that more than half of their survey respondents did not believe in the clinical efficacy of EUS for managing patients with gastric cancer. They also reported a trend toward underutilization of EUS when staging gastric cancer, which was unaffected by the availability of EUS in practice. At present, there are no guidelines for indicating EUS examination. EUS guidelines must be established to increase the accuracy of EUS and expand its use.

## Conclusions

The accuracy of T staging by EUS appeared to decrease with detailed T staging. T overstaging, which caused decreased accuracy, was correlated with lesions greater than 50 mm, and incorrect EUS nodal staging was associated with larger tumor size and deeper tumor infiltration. Consequently, careful EUS examination in association with other diagnostic tools must precede treatment planning for gastric cancer with these characteristics. Improvement in EUS equipment and techniques will be essential to overcome the weak points of the method, and an implementation guideline should be developed for improvement of the clinical efficacy of EUS.

## Competing interests

The authors have no conflicts of interest or financial ties to disclose.

## Authors’ contributions

HHL and CHP were involved in the conception and design of the study, carried out data analysis, and drafted the manuscript. CHL, JMP and YKC were responsible for acquisition and interpretation of data.KYS, and HMJ were responsible for interpretation of data and CHP, KYS and HMJ carried out critical revision of the manuscript. All authors have read and approved the final manuscript.
